# Modeling the impact of the habituation effect on information spreading processes with repeated contacts under an SI model

**DOI:** 10.1371/journal.pone.0280266

**Published:** 2023-04-12

**Authors:** Piotr Bartków, Kamil Bortko, Jarosław Jankowski, Patryk Pazura

**Affiliations:** West Pomeranian University of Technology, Faculty of Computer Science and Information Technology, Szczecin, Poland; Unviersity of Burgundy, FRANCE

## Abstract

People are exposed to information from different sources whether or not such exposure is desired. Due to a limited ability to process information, only parts of the messages may be absorbed, and other parts may be ignored. Repeated stimuli lead to lower responses due to the learning process and the habituation effect. While this effect has been intensively studied, mainly in relation to visual stimulus, it is also incorporated in information spreading processes. Information spreading models often assume the possibility of repeated contact, but no habituation effect, which lowers the level of responsiveness of nodes in the network, has been implemented. Here, we study the impact of the habituation effect on information spreading with a susceptible–infected (SI) model, which is often the basis for other models. We assume that a decrease in habituation has an impact on propagation processes. Analysis of the results shows that the course of these propagation processes behave differently, significantly worsening their results. These processes are very sensitive, even to small changes in the level of habituation.

## 1. Introduction

Electronic media are used extensively for marketing operations, often leading to marketing content overload. Users experience advertising clutter with many commercial messages delivered within portals and social media [1]. The perceived intrusiveness of marketing content may negatively impact brand awareness, and overall performance [2]. It often leads to advertising avoidance within social media and the usage of ad blocking software [3]. From the perspective of perception and a limited ability to process information, content is filtered, and only a limited number of messages is absorbed [4]. In the area of visual advertising, banner blindness is resulting in ignored marketing content within visual spaces [5, 6].

The habituation effect is one of the reasons for reduced response [7]. It was initially analyzed from the perspective of biological systems and can be understood as a form of basic learning [8]. While the habituation effect was identified and studied in the 1960s [9], new goals and directions have been identified, including new ways of separating habituation from sensory adaptation or fatigue [10].

Apart from empirical experiments, the need for new predictive models is emphasized [11]. Even at an early stage of research, simulation models were used for modeling synaptic mechanisms [12]. Differential equations from first applications [13] were extended to model inter-stimulus intervals [14] or the impact of long-term memory [15].

Apart from biological systems, habituation was taken into account in artificial systems and robots [16]. The purpose of the model is to represent visual attention for computer programs or robots [17]. Novelty detection algorithms were also inspired by habituation studies [18]. Applications of habituation mechanisms to machine-learning processes were implemented to make the learning process closer to biological systems, because, even in such case, a decrease in the responsiveness of the learning process has been observed over time [19]. Models of habituation were also used for multi-armed bandits algorithms for marketing online content delivery optimization [20]. Recent efforts were related to predictability [21], visual stimulus [22], and modeling emotional habituation [23].

Besides visual communication and display advertising, repeated stimuli are also typical for word-of-mouth actions within real or digital social networks. The social network structure allows for the flow of various kinds of content. This can be any information, idea, visual content, or viral movie. Social network members perceive repeated exposures, and its impact on information spreading was analyzed [24]. Repetitions can deliver a cumulative impact on consumer behavior and increase the probability of purchase [25]. This results in the extension of the influence maximization problem towards repeated contacts. The cumulative influence was also analyzed for threshold models, and pieces of information received by users in each step are accumulated before the final decision takes place [26]. Multiple received signals were used as an extension of single activation models to reach threshold zones [27]. Analogies can be found in epidemiology research, where transmission probability is related to a number of contacts with an infected person [28]. Smieszek et al. focused on contact repetition and proposed an extension of the SIR model [29]. Earlier, the deterministic epidemic model taking into account repeated contacts was proposed, and the repetition impact of spreading effectivity was analyzed [30]. Repetitions of periods of partnership contact are also typical for sexually transmitted diseases [31].

While various models are used to represent behaviors based on repeated messages, the drop in response after repeated messages was not taken into account. Most models assume that repeated massages will not decrease the effectiveness of the spreading process but rather increase. While a cumulative impact can increase performance, from another perspective, repeated communication can be perceived as unwanted, and the probability of purchases can generally decrease [32]. Incentivized viral campaigns generate a high number of repeated contact, and the performance represented by conversion rate can decrease [33]. Intensive informational campaigns focused on changes in social behavior, for example, changes in behavior during a pandemic, are performed [34]. They do not always deliver the expected results, and habituation can be one of the reasons for a decreased response.

In terms of spreading within networks, the habituation effect was modeled earlier under an Independent Cascades Model [35]. The IC model proposed in [36] assumes that repeated contacts are observed only when communication with the same content comes from other different users. A single user has only one chance to activate other user, so repetition between two users never exists.

In the current study, we focused on a situation that is more common for information spreading: when a repeated message can flow between the same nodes. We adopted a susceptible–infected (SI) epidemic model, which has been discussed and used for information spread in many studies [37, 38]. In the basic SI model, a failed attempt to transmit information or a virus does not affect the probability of success of subsequent attempts. The difference in our model is that, as a result of the habituation process, each failed attempt lowers the chances of infection in the next step. It assumes repetitions between the same users, which represents a situation when several attempts to deliver a message are taken between two network members. The habituation effect was integrated within the model, and experiments were performed for different parameters of response decrease.

## 2. The habituation effect and its integration within the susceptible–infected (SI) model

Habituation is a cognitive process that involves the fading of a response to a stimulus [11]. In practice, it can be assumed that, as a species, we are generally able to adapt to changes that occur. We react to a variety of different external stimuli with the senses. As time goes by, when the stimulus no longer causes the same reaction or simply does not change, the reaction fades. At the beginning of 2020, we faced two major events that may in some way illustrate the fading of reactions and the “taming” of society to the changes that took place. The first is the COVID-19 epidemic, and the second is Russia’s attack on Ukraine. It was possible to observe how the amount of information, or, unfortunately, disinformation, on a given topic can increase. Eastern European countries experienced several disinformation attacks when the war began, aimed at weakening the mobilization of the population in providing aid to refugees. This was partially successful because of the strong emotional appeal of this content, fueled by the natural fear of war. Border countries were particularly vulnerable to attacks because of its assistance to refugees. They have experienced two major panic attacks in society, fueled by so-called “trolls” on social media. This is an example of how habituation can work to the advantage of the recipient, weakening their reaction to acutely harmful message. Fear is never a good advisor, and it most easily intensifies the user’s reaction. At the beginning of the war, information about sudden increases in fuel prices caused panic and queues. The result was a threat of fuel shortages for special forces vehicles. The second situation concerned a shortage of sugar. The panic caused people to buy up supplies, and the stores ran out of sugar for two weeks. Both situations threatened a direct decline in the quality of life, which resulted in a psychological attack on a large part of the population.

In the middle of these events the idea was born about conducting a study on how habituation impacts the way various content spreads through social networks. In this study we make no distinction between the spread of information or contagion; nor do we specify the exact type of information in question, as the entirety of humanity’s developement has occured throught the mechanisms of information spread. It is irrelevant if what is being spread is religion, disease, fashion, lifestyle, ideology, or the elementary skills like the ability to write or basic math. It could even be an emotion, like outrage or fear; in fact we’ve seen this year how fear of war spreads, and despite the importance and gravity of this subject we may already see a decline of interest and strength of response to the ongoing conflict. That’s habituation, and we are interested exclusively in how this process affects the spread of our test outbreak.

**Fig 1:**
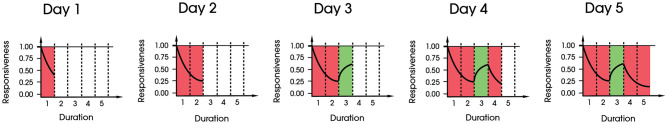
Example illustrating the impact of repeated ineffective/unwanted contacts on node responsiveness.

This section presents the idea of integration habituation with the spreading model. Fig 1 shows how responsiveness level curve is shaped by the effect of habituation. Imagine having a friend, who is trying to convince you to their point of view at almost every opportunity. During first two meetings an attempt to spread this information occurs, with the subject, namely us, showing no positive response. On the contrary, our level of responsiveness decreases, while our irritation rises. During third meeting there is no spread attempt; it might be a simple conversation about the weather, but whatever the subject, it gives us a welcome break from the annoying content and our level of responsiveness to the subject rebounds. During the next two meetings, however, further attempts occur. This results in our responsiveness falling to almost zero. The interpretation is that it’s not simply that we don’t want to listen, it’s that at this point we don’t want to even meet our troublesome friend.

The SI model generates a very large number of contacts, each one resulting in an attempt to transmit information. This results in a faster coverage process compared to SIR or SIRS models, where nodes can undergo “healing.” We found that it will be best suited to test the effectiveness of multiple contacts. This situation can generate a large number of unsuccessful attempts, which cause a decrease in responsiveness with each successive attempt.

In our study, we assume that each node wants to provide the same information. What kind of information it is is not important, because any content under certain conditions can be tiresome. For this reason, in our model, the studied epidemics have fixed parameters, while the edge weights are randomized for each individual contact in the network. As in real life, we have various moods or coincidences from different people under certain conditions, and messages may be more digestible.

The SI model allows infected nodes to contact their neighbors as many times as possible. They are not able to recover from infection like in other, previously mentioned models. For this reason, the situation, as shown on Day 3 in Fig 1, will not have a chance to occur. In the SI model, if an infected node appears in the neighborhood of a node, it will attempt to infect the susceptible node in each successive simulation step until it succeeds. In our assumption, each failed attempt will affect the probability of propagation in each subsequent attempt. Of course, a node can become infected on the first attempt, in which case its level of responsiveness will not change. A song can be “catchy” upon hearing it for the first time, and some may attest to the experience of “love at first sight.” Arguably, an interesting assumption would be that the level of responsiveness at which we adopt a given piece of content influences the “fervor” with which we will propagate it further. However, this could already be a different kind of responsiveness, since every stimulus we interact with affects our resistance to a variety of factors.

At the beginning of each simulation, the infection process begins with a group of nodes. In the first step, each infected node gets one chance each to infect susceptible neighbors. Nodes having several active neighbors that will be contacted several times in one turn. A node infected in a given queue is added to the pool of seeders and will be able to infect a neighbor in the next step. Nodes that are not infected after contact have their responsiveness level lowered, which decreases the chance of success in the next attempt. This process will continue until the network is fully covered. In our study, we used a coverage threshold of 80%. We chose this limit because of the significant decrease in process dynamics and the time required for 100% coverage.

**Fig 2:**
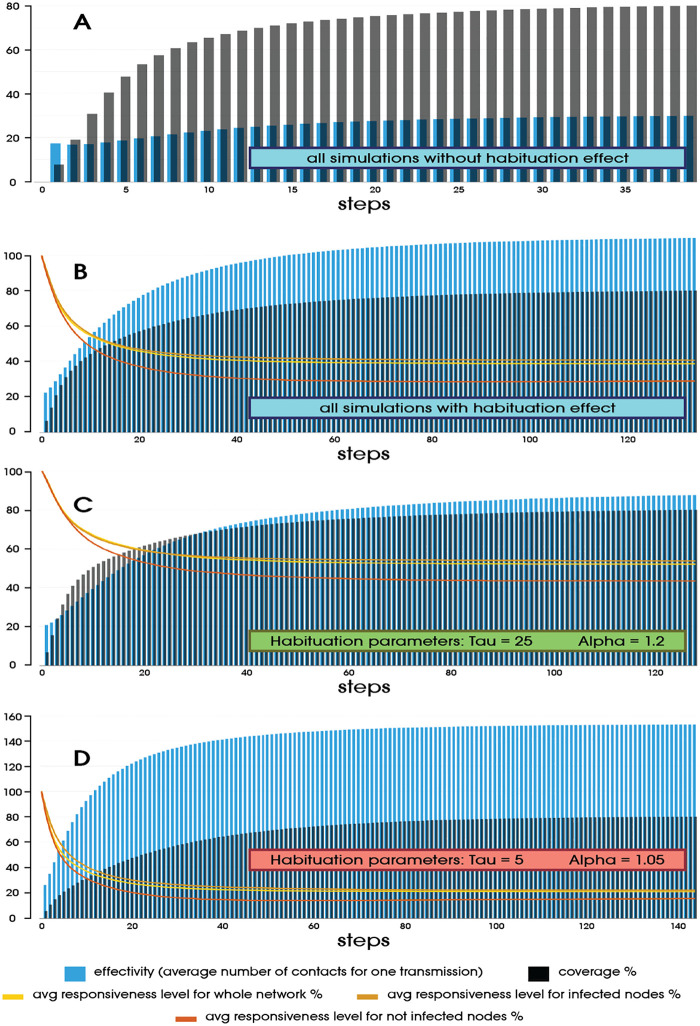
Example illustrating the impact of a drop in responsiveness on process performance.

Fig 2 shows how the dynamics of network coverage evolves. Graph 2.A shows the average of all runs without the effect of habituation. These processes took 40 simulation steps to reach the threshold, with an average of about 20 attempts per successful infection. Chart 2.B, on the other hand, shows the averaged runs for the same simulations, already showing a decrease in responsiveness. It took 100 contacts to infect one node, making the time increase by more than three times. Plot 2.C and 2.D show the results for the most and least favorable habituation parameters, respectively. Overlaid on all graphs having the effect of habituation are curves of decreasing responsiveness for all nodes, infected nodes, and uninfected nodes, which includes those not yet contacted.

In the next section, we describe in more detail the assumptions of the experiment, the parameters used, and the computational method.

## 3. Experiment setup and the mathematical method

Agent-based simulations were performed on four real networks. All were from public repositories. The parameters of the chosen networks are presented in Table 1.

**Table 1:** Main network characteristics for Networks N1–N4, including the number of nodes and edges, the mean degree (DG), network density (ND), global clustering coefficient (CC), mean eigenvector centrality (EV), and modularity (MD).

Networks	Source	Nodes	Edges	DG	ND	CC	EV	MD	Reference
N1	University of California	899	7019	16.62	0.0174	0.07	0.14	0.22	[39]
N2	Political blogs	1224	16715	27.31	0.0223	0.23	0.1	0.43	[40]
N3	Hamsterster friendships	1858	12534	13.49	0.0073	0.09	0.05	0.45	[41]
N4	UoCalifornia messages	1899	13838	14.57	0.0077	0.06	0.08	0.26	[42]

Experiments were simulated within the given network *N*(*V*, *E*) based on the vertex set $V=v_{1},v_{2},\ldots ,v_{m}$ and edge set $E=e_{1},e_{2},\ldots ,e_{n}$. Simulations were performed using the proposed Susceptible–Infected model. Each node *u* ∈ *V* has a relationship represented by an edge (*u*, *v*)∈*E*. At each step *t*+ 1 of the simulation, every node *v* ∈ *V* can be infected by his neighbor with a propagation probability *PP*(*u*, *v*) provided that the infecting neighbor was infected in step *t* < *t* + 1.

In this study, we focused on investigating the impact of habituation on network coverage and infection duration. For the purpose of the experiment, a test space was created, consisting of the following parameters, *R* × *N* × *PP* × *SP* × *H* × *A* × *T* with a seeding strategy based on single-stage seeding. This provided us with 640 combinations, each of which was performed 10 times, which allowed us to analyze the influence of individual parameters on the course of the infection process. Randomized probabilities were used for each simulation, which was drawn at each attempt to infect a susceptible node on a given edge. Details of the parameters used are given in Table 2.

**Table 2:** Parameters used for diffusion in the simulations.

Symbol	Parameter	Variants	Values
R	Ranking Type	2	Random, Degree
N	Network	4	N1, N2, N3, N4
PP	Propagation probability	2	0.05, 0.1
SP	Seed fraction	2	1%, 5%
H	Habituation	2	Exists, Not exists
A	*α*	2	1.05, 1.2
T	*τ*	5	5, 10, 15, 20, 25

Each simulation begins with a group of activated nodes Φ(*s*_0_) in a given graph *G*(*V*, *E*). In each subsequent simulation step *s*, a set of nodes Φ(*s* − 1) activated at step *s* − 1 is generated before the contagion process begins. For each node from the set *u* ∈ Φ(*s* − 1), a list of susceptible neighbors is created *Θ*(*v*, *s*). For each node *v* ∈ *Θ*(*v*, *s*), activation is attempted by node *u*. Activation occurs when the randomly generated number on the edge between the nodes concerned is lower than the given *PP*(*u*, *v*). Propagation probability is equal for all steps. If the activation attempt is successful, the newly infected node migrates to the set of nodes infected in this step Φ(*s*) and will be able to participate in the infection process in the next step *s* + 1 as a spreader.

Due to the integration of the model with the habituation effect, each node is also assigned a responsiveness level *R*(*v*, *s*), which is used to calculate the node-specific propagation probability at a given simulation stage. Responsiveness decreases due to a failed contagion attempt, which directly affects the probability of propagation according to our model. If *R*(*v*, *s*)<1.0, then, for a given *PP*_*s*_, its new value for a given node is calculated according to the formula *PP*_*s*_(*u*, *v*, *s*) = *PP*(*u*, *v*) * *R*(*v*, *s*).

The calculation of the responsiveness factor for a given node is performed after each contact. At the beginning of each simulation step, nodes can be in one of two states: 1, active, or 0, inactive. Active nodes may attempt to infect. Each contact in the network can result in one of two possibilities: ineffective activation (unwanted messages) or activation. When a node is activated, the level of responsiveness does not change, and such a node already functions as a spreader.

When an unsuccessful attempt is made, responsiveness is calculated according to Formula (1):

$$\begin{array}{c} y=y_{0}-\frac{S}{\alpha }\left(1-\text{exp}\left(\frac{\alpha \cdot Cnt_{+1}}{\tau }\right)\right) \end{array}$$ (1)

where *y*_0_ represents the initial habituation value. For non-contacted inactive nodes, it is 1.0; for inactive contacted nodes, it is valid for each discreet time point *t*. *S* represents stimulus exposition and in this experiment always takes the value of 1 because of the number of actions in the current time step. *α* is responsible for the recovery rate. *τ* is a constant influencing habituation process. *t* is valid for the time that has passed since responsiveness began to drop.

An increase in responsiveness in the SI model can occur when there is no interaction between nodes. As we have mentioned, there are no interruptions in the SI model, so the following method was added for the universality of the algorithm and in terms of future research. Growth can be calculated using Formula (2):

$$\begin{array}{c} y=y_{0}-\left(y_{0}-y_{1}\right)\text{exp}\left(\frac{-\alpha \cdot Cnt_{-1}}{\tau }\right) \end{array}$$ (2)

where *y*_0_ represents the initial responsiveness value, equal to 1.0, *y*_1_ refers to the responsiveness value reached during the decrease periods, and *t*, in this case, represents the time passed from the beginning of the recovery process.

In both cases, time t does not represent simulation steps, but the number of contacts. In our study, the reason for the decrease in responsiveness is each failed message delivery attempt, not the time in the sense of a simulation step in which there is a variable number of attacks per node.

## 4. Illustrative example

**Fig 3:**
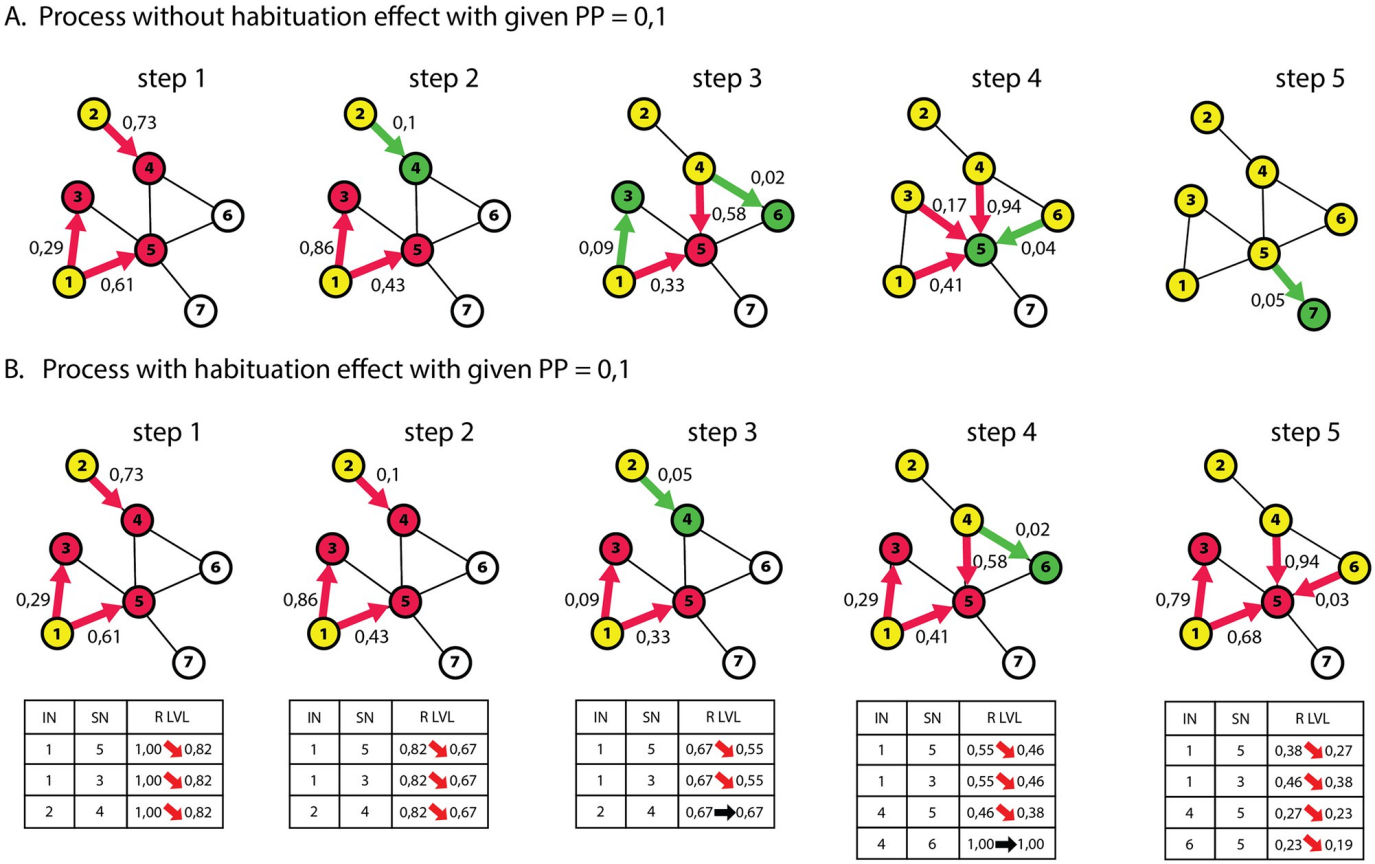
Toy example showing the steps of the simulation for processes with and without the habituation effect.

In the following section, we will present a simplified process and how it differs from the basic SI model. The network slice shown consists of seven nodes connected by eight edges. Fig 3 is divided into two parts: A, corresponding to the process without the effect of habituation, and B, which assumes this effect. Both show five steps of the simulation. For both processes, the assumed propagation probability threshold is 0.1. In turn, the edge weights are randomized for a given edge at each successive contact. Each contact results in an attempt to pass the content on. In the case of Process 3.A, contacts resulting in failed attempts do not reduce the propagation probability in any way. For 3.B, each node is assigned an initial responsiveness level of 1. This level is reduced based on Formula (1) from the previous section. Under the figures with each step, there are tables with the current responsiveness levels (R LVL) and information about which relationship is affected (IN: infected node; SN: susceptible node). The probability of propagation is always multiplied by the current level of responsiveness of a given node. Infection occurs when the drawn edge weight is less than or equal to the assumed propagation probability. Selected parameters of the habituation process are *τ* = 5 and *α* = 1.05.

In Step 1, both Process A and B fail to infect any new node because the drawn edge weights do not allow it. However, in a process with the habituation effect superimposed, each ineffective contact resulted in a reduction in the levels of responsiveness, which are shown in the table under Fig 3.A.step1. This means that each attack in Step 2 will take place not with an assumed threshold of 0.1 but with a threshold of 0.1 X 0.82 = 0.082.

Step 2 marks the first time that a decrease in responsiveness affects the process. The drawn edge weight is equal to the assumed threshold, so in Fig 3.A.step2, the activation of Node 4 by Node 1 occurs. In Example Fig 3.B.step2, contagion will not occur because the responsiveness level will lower the assumed activation threshold to 0.082 as a result of a previous failed attempt. Since no new nodes have been infected, we know that, in the next round, all nodes will have their responsiveness reduced to 0.67, resulting in a PP of 0.067 for them instead of the given 0.1.

For a process with no habituation effect, two more infections and one ineffective contact occur in Step 3. The process with the superimposed effect succeeds in infecting the first node. Despite the reduced PP, the drawn weight is smaller: 0.06 is smaller than 0.1 reduced to 0.067. In Fig 3.B.step3, on the edge between Node 1 and 3, a situation like in Fig 3.B.step2 occurs between Node 2 and 4. The assumed threshold without reduced responsiveness would allow contagion on this edge as in Fig 3.A.step3 between the same nodes.

In Step 4, one infection occurs in both examples. In Fig 3.B.step4, infection occurs between Node 4 and Node 6. This happens at the first contact; as a result of this, the level of responsiveness of Node 6 does not change. As we mentioned in an earlier chapter, once infected, the level of responsiveness of such a node has no effect on whether it will pass the information on.

In Fig 3.A.step5, the last vulnerable node is infected. At the same stage, the process with the habituation effect covered less than 60% of the network. In addition, Node 5 has its responsiveness level reduced to 0.19. Such a node can only be infected if the minimum activation threshold at the next contact is method.

The graphic above shows how each successive contact generating another opportunity affects the length of the process. This can be understood as the cost of the next attempt. It is possible to try one more time to reach the customer, but this generates additional costs and can cause the opposite reaction, i.e., discouragement or exhaustion with the amount of content delivered.

## 5. Results

The main objective of this study was to integrate the effect of habituation within the model of information spreading processes and analyze its effect on the performance of spreading processes. The SI model was used as one of the basic models with many extensions and based on the repeated contact between nodes. It was initially proposed for the spread of epidemics and has also been implemented for information diffusion. The differences in the process with and without the habituation process were analyzed. Simulations were performed on the agent-based proposed model with the integration of the habituation process. All processes had the target of achieving a network coverage level of 80%. This decision was made to standardize the results, as when testing the longest intervals, the processing time needed for full coverage increased significantly, as will also be shown in the analysis below. The experiment was based on different parameters such as ranking types, networks, propagation probabilities, seed fraction characteristics for spreading models, and the habituation process parameters *τ* and *α*. All results for the individual parameters can be found in the Tables 3–6. In order to highlight the characteristics of the influence of the habituation effect on the spreading process, we decided to divide the graphs into two sections: network coverage and process duration. The network coverage section presents the results for each considered parameter after 5, 10, and 15 simulation steps. Process duration presents the values for half of the assumed threshold, in this case 40%, and after reaching the goal, i.e., infecting 80% of the network.

**Table 3:** Coverage for spreading processes with and without habituation.

Parameter	Value	HAB						NO HAB		
		5 steps	p-value	10 steps	p-value	15 steps	p-value	5 steps	10 steps	15 steps
		43.74%	<2.2e-16	59.79%	<2.2e-16	66.77%	<2.2e-16	62.91%	78.55%	80.00%
NET	N1	42.74%	<2.2e-16	59.98%	<2.2e-16	67.09%	<2.2e-16	62.79%	79.81%	80.00%
	N2	52.75%	<2.2e-16	67.12%	<2.2e-16	72.71%	<2.2e-16	73.12%	80.00%	80.00%
	N3	37.93%	<2.2e-16	54.60%	<2.2e-16	62.37%	<2.2e-16	55.86%	76.04%	80.00%
	N4	41.53%	<2.2e-16	57.45%	<2.2e-16	64.93%	<2.2e-16	59.84%	77.02%	80.00%
ALPHA	1.05	42.21%	<2.2e-16	57.41%	<2.2e-16	64.56%	<2.2e-16	62.91%	78.55%	80.00%
	1.2	45.26%	<2.2e-16	62.16%	<2.2e-16	68.99%	<2.2e-16	62.91%	78.55%	80.00%
PP	0.05	31.59%	<2.2e-16	48.25%	<2.2e-16	57.47%	<2.2e-16	53.91%	75.67%	80.00%
	0.1	55.89%	<2.2e-16	71.32%	<2.2e-16	76.08%	<2.2e-16	71.90%	80.00%	80.00%
SP	0.01	41.41%	<2.2e-16	59.59%	<2.2e-16	67.06%	<2.2e-16	60.25%	78.70%	80.00%
	0.05	46.06%	<2.2e-16	59.98%	<2.2e-16	66.49%	<2.2e-16	65.56%	78.39%	80.00%
TAU	5	31.36%	<2.2e-16	45.60%	<2.2e-16	54.35%	<2.2e-16	62.91%	78.55%	80.00%
	10	40.64%	<2.2e-16	56.20%	<2.2e-16	63.92%	<2.2e-16	62.91%	78.55%	80.00%
	15	59.92%	<2.2e-16	62.31%	<2.2e-16	69.19%	<2.2e-16	62.91%	78.55%	80.00%
	20	49.29%	<2.2e-16	66.22%	<2.2e-16	72.31%	<2.2e-16	62.91%	78.55%	80.00%
	25	51.47%	<2.2e-16	68.61%	<2.2e-16	74.10%	<2.2e-16	62.91%	78.55%	80.00%

**Table 4:** Duration for spreading processes with and without habituation.

Parameter	Value	HAB				NO HAB	
		40% coverage	p-value	80% coverage	p-value	40% coverage	80% coverage
		6.15	<2.2e-16	39.83	<2.2e-16	3.30	9.46
NET	N1	6.33	<2.2e-16	33.14	<2.2e-16	3.51	8.5
	N2	4.27	<2.2e-16	26.48	<2.2e-16	2.58	7.03
	N3	7.52	<2.2e-16	53.33	<2.2e-16	3.71	11.60
	N4	6.48	<2.2e-16	46.37	<2.2e-16	3.40	10.70
ALPHA	1.05	6.53	<2.2e-16	45.40	<2.2e-16	3.30	9.46
	1.2	5.77	<2.2e-16	34.26	<2.2e-16	3.30	9.46
PP	0.05	8.51	<2.2e-16	58.14	<2.2e-16	3.84	11.91
	0.1	3.79	<2.2e-16	21.52	<2.2e-16	2.76	7.01
SP	0.01	6.59	<2.2e-16	35.13	<2.2e-16	3.76	9.16
	0.05	5.71	<2.2e-16	44.53	<2.2e-16	2.84	9.76
TAU	5	9.89	<2.2e-16	67.42	<2.2e-16	3.30	9.46
	10	6.55	<2.2e-16	46.84	<2.2e-16	3.30	9.46
	15	5.27	<2.2e-16	34.84	<2.2e-16	3.30	9.46
	20	4.66	<2.2e-16	27.23	<2.2e-16	3.30	9.46
	25	4.38	<2.2e-16	22.82	<2.2e-16	3.30	9.46

**Table 5:** Coverage decrease for spreading processes with the habituation effect.

Parameter	Value	5 steps	p-value	10 steps	p-value	15 steps	p-value
NET	N1	31.93%	<2.2e-16	24.85%	<2.2e-16	16.14%	<2.2e-16
	N2	27.86%	<2.2e-16	16.10%	<2.2e-16	9.11%	<2.2e-16
	N3	32.10%	<2.2e-16	28.20%	<2.2e-16	22.04%	<2.2e-16
	N4	30.60%	<2.2e-16	25.41%	<2.2e-16	18.84%	<2.2e-16
ALPHA	1.05	32.90%	<2.2e-16	26.91%	<2.2e-16	19.30%	<2.2e-16
	1.2	28.06%	<2.2e-16	20.87%	<2.2e-16	13.76%	<2.2e-16
PP	0.05	41.40%	<2.2e-16	36.24%	<2.2e-16	28.16%	<2.2e-16
	0.1	22.27%	<2.2e-16	10.85%	<2.2e-16	4.90%	<2.2e-16
SP	0.01	31.27%	<2.2e-16	24.28%	<2.2e-16	16.17%	<2.2e-16
	0.05	29.74%	<2.2e-16	23.49%	<2.2e-16	16.89%	<2.2e-16
TAU	5	50.15%	<2.2e-16	41.95%	<2.2e-16	32.06%	<2.2e-16
	10	35.40%	<2.2e-16	28.45%	<2.2e-16	20.10%	<2.2e-16
	15	27.01%	<2.2e-16	20.67%	<2.2e-16	13.51%	<2.2e-16
	20	21.65%	<2.2e-16	15.70%	<2.2e-16	9.61%	<2.2e-16
	25	18.18%	<2.2e-16	12.65%	<2.2e-16	7.38%	<2.2e-16

**Table 6:** Duration increase for spreading processes with the habituation effect.

Parameter	Value	40% coverage	p-value	80% coverage	p-value
NET	N1	80.34%	<2.2e-16	289.88%	<2.2e-16
	N2	65.50%	<2.2e-16	276.67%	<2.2e-16
	N3	102.70%	<2.2e-16	359.74%	<2.2e-16
	N4	90.59%	<2.2e-16	333.36%	<2.2e-16
ALPHA	1.05	97.88%	<2.2e-16	379.92%	<2.2e-16
	1.2	74.85%	<2.2e-16	262.16%	<2.2e-16
PP	0.05	121.61%	<2.2e-16	388.16%	<2.2e-16
	0.1	37.32%	<2.2e-16	206.99%	<2.2e-16
SP	0.01	75.27%	<2.2e-16	283.52%	<2.2e-16
	0.05	101.06%	<2.2e-16	356.25%	<2.2e-16
TAU	5	199.70%	<2.2e-16	612.68%	<2.2e-16
	10	98.48%	<2.2e-16	395.14%	<2.2e-16
	15	59.70%	<2.2e-16	268.29%	<2.2e-16
	20	41.21%	<2.2e-16	197.84%	<2.2e-16
	25	32.73%	<2.2e-16	141.23%	<2.2e-16

## Impact of the habituation effect on network coverage in information-spreading processes

**Fig 4:**
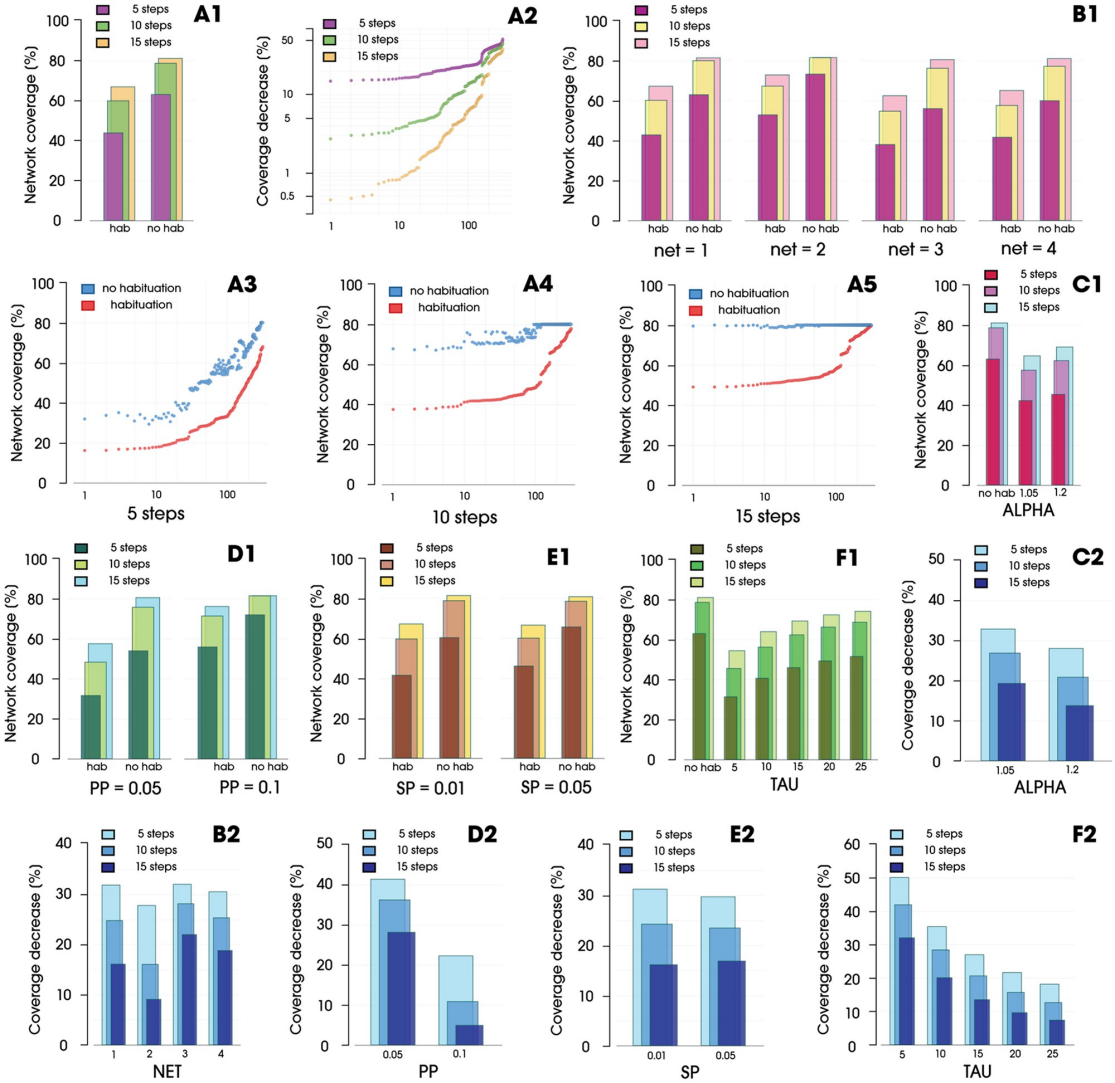
(**A1**) Coverage for spreading processes with and without habituation. (**A2**) Coverage decrease in processes with the habituation effect, compared to processes without habituation, sorted by the decrease in coverage. (**A3**--**A5**) Distances between coverage in simulations with and without the habituation effect, with results sorted by coverage without habituation and assigned corresponding results from processes with the habituation effect. (**B1**) Coverage for each network for spreading processes with and without habituation. (**B2**) Decrease in coverage for each network in relation to a process without habituation. (**C1**) Coverage for each alpha value for spreading processes with habituation compared to a process without habituation. (**C2**) Decrease in coverage for each alpha in relation to a process without habituation. (**D1**) Coverage for propagation probabilities for spreading processes with and without habituation. (**D2**) Decrease in coverage for each PP value in relation to a process without habituation. (**E1**) Coverage for each seeding percentage for spreading processes with and without habituation. (**E2**) Decrease in coverage for each SP in relation to a process without habituation. (**F1**) Coverage for each tau value for spreading processes with habituation in comparison with processes without habituation. (**F2**) Decrease in coverage for each tau in relation to a process without habituation.

The average results for all simulation runs with all parameters for processes with and without the habituation effect are presented in Fig 4.A1. It can be clearly seen that, at each simulation run presented, the process with habituation performed relatively poorly. After five steps, the difference in favor of the process without habituation was 19.17%. After 10 steps, it decreased slightly and amounted to 18.76%. After 15 steps, the process without habituation reached the intended 80%, and its advantage over the “chasing” process with the habituation effect was equal to 14.2%. Parameters showed statistical significance. The Wilcoxon test showed p-values less than 0.05 for both *τ* and *α*. Fig 4.A2 shows the drops in coverage for all processes with habituation, sorted by the drop in coverage. It can be seen that the longer the simulation lasted, the smaller the decreases were, which was due to the fact that some processes did not even need seven steps to reach the target. The largest difference after five steps was 52.2%. After 10 steps, we observed a value of 45.51%; after 15 steps, it was 39.4%. The smallest differences also decreased over time, with 15.05%, 2.65%, and 0.28% after 5, 10, and 15 steps, respectively. Fig 4.A3 to 4.A5 shows all the runs sorted by the coverage of the processes without habituation. Each graph represents the situation after 5, 10, and 15 steps.

**Fig 5:**
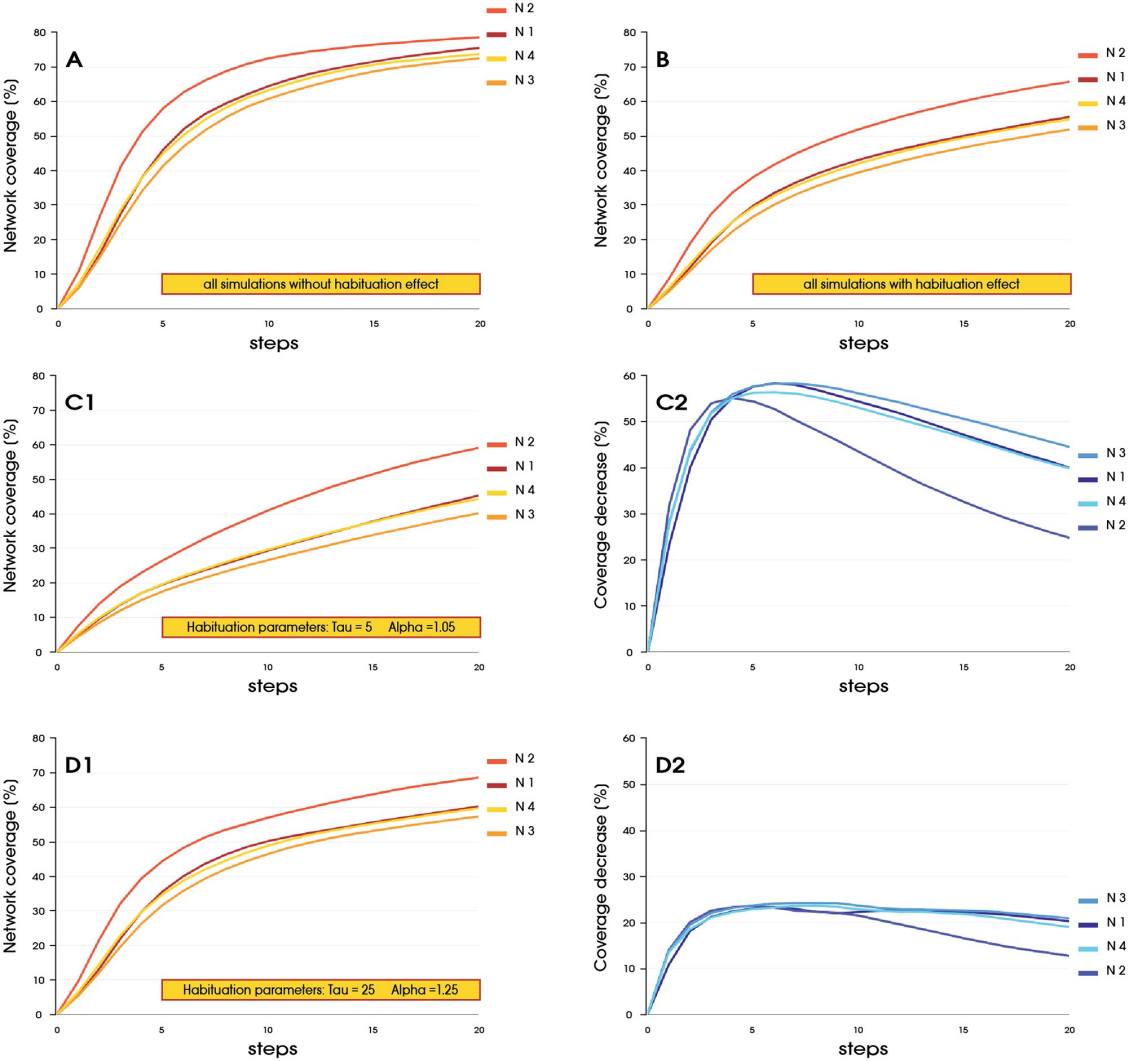
(**A**) Coverage for each network for all spreading processes without habituation. (**B**) Coverage for each network for all spreading processes with habituation. (**C1**) Coverage for each network for spreading processes with the “worst” set of habituation parameters. (**C2**) Decrease in coverage for each network in relation to a process without habituation with the “worst” habituation parameters. (**D1**) Coverage for each network for spreading processes with the “best” set of habituation parameters.(**D2**) Decrease in coverage for each network in relation to a process without habituation with the “best” habituation parameters.

Considering the individual networks used for the simulations (Fig 4.B1), it can be seen that, when simulations without the effect of habituation performed better on a given network correspondingly, processes with applied habituation also showed improved performance compared with the other runs for the other networks. *Net*2 obtained the best results for both types of processes. It has the highest mean degree (DG = 27.31), clustering coefficient (CC = 0.23) and a network density of two to three times that of the other networks (ND = 0.0223—see Table 1). The worst effects were observed for *Net*3. Comparing these extreme cases, we obtained 52.27% for *Net*2 and 37.93% for *Net*3 after 5 steps, and these values were 67.12% and 54.60% after 10 steps and were 72,71% and 62,37% after 15 step, respectively. Fig 4.B2 shows the percentage drops in coverage at the selected steps. For Network 2 and 3, the difference in coverage in favor of Network 2 was 4.24% after 5 steps, 12.1% after 10 steps, and 12.93% after 15 steps. Thus, looking at the specifics of the network, it can be concluded that the network density became more important over time in terms of reducing the effect of habituation. When the average response rate was already strongly reduced for the whole network, the number of connections became more important. The model used, makes the number of possible. contacts the most important element affecting the speed of coverage. The impact of network heterogeneity is further shown in the Fig 5. Parameters such as modularity, eigenvector do not show significant interference with the processes within set of analysed networks. Fig 5.A and 5.B shows all runs for selected networks with and without the imposed habituation effect. Fig 5.C1 and 5.D1 presents the “worst” and the “best” set of habituation parameters, respectively. Fig 5.C2 and 5.D2, on the other hand, show declines in the performance of these processes relative to simulations without the habituation effect applied.

Fig 4.C1 illustrates the comparison between simulations without habituation and simulations in terms of the *α* parameter. This parameter affects the way the response curve flattens out. The higher the value, the lower the decreases are and the higher the increases are during the recovery of a node. This results in minimally improved network coverage results for *α* = 1.2. Fig 4.C2 shows the decreases in network coverage with respect to the process without habituation with respect to the analyzed *α* parameters. The difference in subsequent simulation steps were 4.84%, 6.04%, and 5.54% in favor of the parameter *α* = 1.2.

In Fig 4.D1, we compare the individual propagation probabilities with and without the implemented habituation effect. In this case, there was a clear difference in favor of *PP* = 0.1. A higher propagation probability resulted in faster network coverage. Fig 4.D2, on the other hand, shows that, comparing the processes with the habituation effect, a higher *PP* also resulted in smaller drops compared to the processes without the effect of habituation; thus, after 5, 10, and 15 simulation steps, the difference in favor of *PP* = 0.1 was, respectively, 19.13%, 25.39%, and 23.26%. At the same time, in the last step in both simulations without habituation, the processes had already reached the assumed 80% coverage, so they did not increase their “advantage”.

The impact of the number of initial nodes initiating the process is shown in Fig 4.E1. It is interesting to note that processes with a higher number of seeds perform worse in terms of network coverage over time. Although the process with *SP* = 0.05 covered 4.65% more of the network after five simulation steps, it was only 0.39% more after 10 steps. After 15 steps, the process with the smaller *SP* (equal to 0.01) achieved a network coverage of 67.06%, whereas the process with the larger SP covered 66.49%, losing 0.57 percentage points. It can be concluded from this that the initial higher number of seeds resulted in an increased number of contacts, as well as the unsuccessful ones, which results in a faster decrease in the level of responsiveness over the whole network, ultimately causing a decrease in the spreading speed. Fig 4.E2, in turn, shows that processes with more grains over time also lose their advantage in terms of a decrease in coverage compared to processes without habituation with the same *SP*. After five steps, the process with a larger *SP* showed a smaller decrease of 29.74%, whereas the processes with a smaller *SP* exhibited a decrease of 31.27%. After 10 steps, they reached 23.49% and 24.28%, respectively. After 15 steps, the situation reversed and the advantage was reached by processes with *SP* = 0.01, losing 16.7%, while processes with *SP* = 0.05 lost 16.89% to processes without the habituation effect.

The second habituation parameter, *τ*, is shown in Fig 4.F1. It is a time constant, and the smaller it is, the more rapidly habituation occurs. This is confirmed in the graphs, as *τ* = 5 achieved the worst result at each stage of the simulation (31.36%, 45.60%, and 54.35%), whereas the largest considered *τ* = 25 covered the network the best (51.47%, 68.61%, and 74.10%). When we look at the declines in coverage shown in Fig 4.F2, we can also notice that, as the *τ* parameter increases, the decline in coverage decreases faster over time relative to processes without habituation with lower values of this parameter.

## Impact of the habituation effect on duration in information-spreading processes

**Fig 6:**
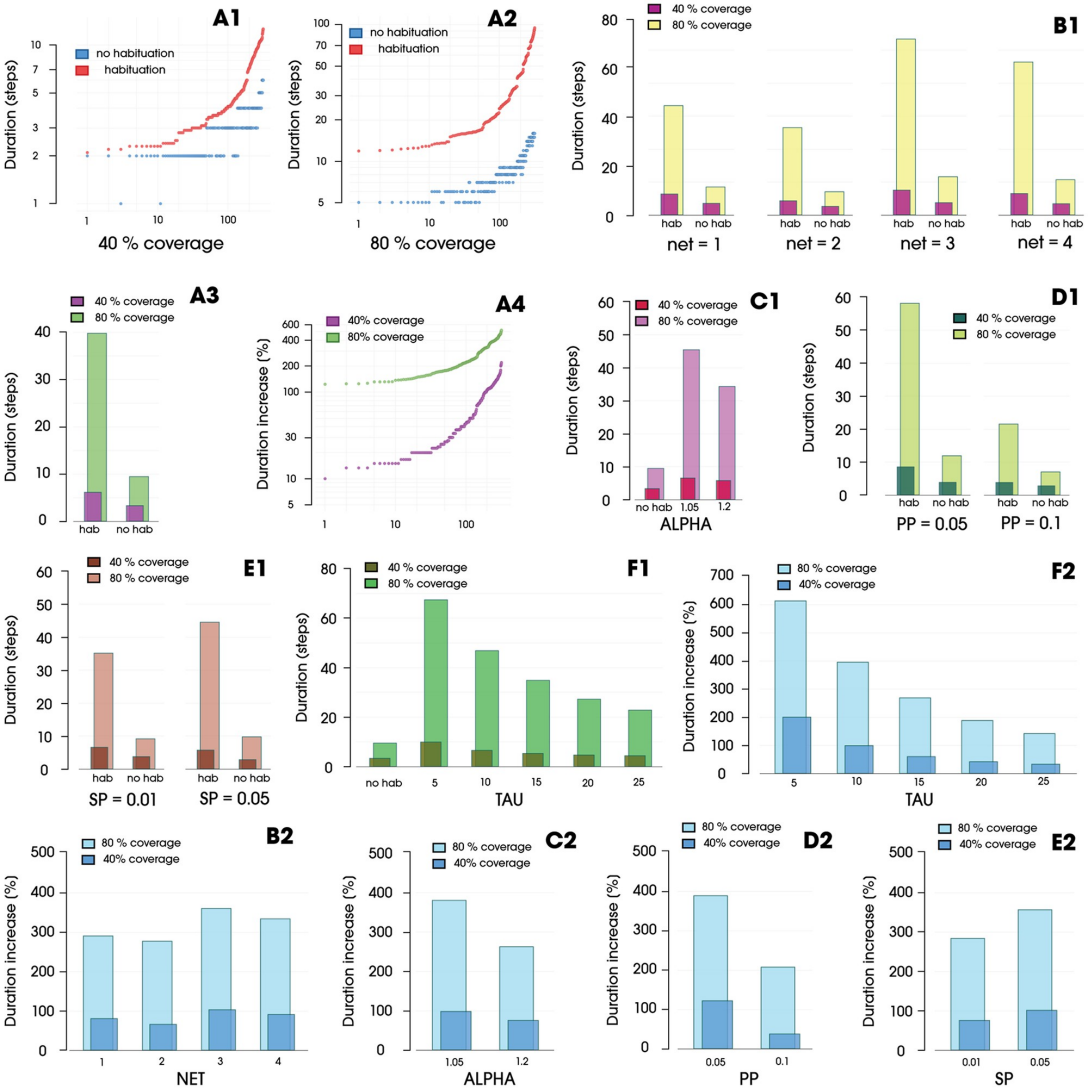
(**A1-A2**) Distances between the durations of simulations with and without the habituation effect, with results sorted by duration without habituation and assigned corresponding results from processes with habituation. (**A3**) Duration for spreading processes with and without habituation. (**A4**) Duration increases in processes with the habituation effect, compared to processes without habituation, sorted by coverage increase. (**B1**) Durations for each network for spreading processes with and without habituation. (**B2**) Increases in duration for each network in relation to a process without habituation. (**C1**) Durations for each alpha value for spreading processes with habituation compared to a process without habituation. (**C2**) Increases in duration for each alpha value in relation to a process without habituation. (**D1**) Durations for propagation probabilities for spreading processes with and without habituation. (**D2**) Increases in duration for each PP in relation to a process without habituation. (**E1**) Durations for each seeding percentage for spreading processes with and without habituation. (**E2**) Increase in duration for each SP in relation to a process without habituation. (**F1**) Durations for each tau value for spreading processes with habituation in comparison with processes without habituation. (**F2**) Increases in duration for each tau value in relation to a process without habituation.

Fig 6.A1 and 6.A2 show all simulation runs divided into processes with and without the implemented habituation effect, sorted in ascending order by length. In both examples, the tendency for the efficiency of the process to decrease (based on its duration) is even more pronounced. A comparison of the averages of all runs can be found in Fig 6.A3. To achieve half of the assumed coverage (40%), processes without habituation required 3.30 simulation steps on average, whereas processes with habituation required almost half as much time (86% more), namely 6.15 steps. Accordingly, for the 80% threshold, the results were as follows: 9.46 steps to 39.83. To achieve the same network coverage, the process with habituation required 321% more time. This was confirmed by the U Mann–Whitney test, which showed the significance (p < 0,05) of the impact of the habituation effect on the duration of the process. For the assumed 40% coverage, the habituation parameters show statistical significance at the level of 0.0466, whereas for the level of 80%, the parameters also showed statistical significance, which was higher, at 0.0098. This confirms the increasing influence of the habituation process on the duration of coverage of the assumed thresholds. Fig 6.A4 compares the magnitude of the duration increases for the earlier graphs (A1, A2). In the case of a 40% network coverage, the increases are in the range of 100%–350%; given an 80% coverage, we obtain a range of duration increases of 250%–650%.

If we look at the division in terms of adopted networks (Fig 6.B1), we can see a division into two groups: *Net*1 with *Net*2, and *Net*3 with *Net*4. The first group has a noticeably higher network density (ND), and degree (DG) values. The second group, on the other hand, is characterized by a significantly higher number of nodes. In the SI model in which the number of contacts is crucial, with low density and the habituation effect implemented, we observe a noticeable decrease in the speed of the processes taking place. The time increases presented in Fig 6.B2 show that, if the processes performed relatively well, they also performed well in relation to “their” collected processes without the habituation effect.

For the comparison of process duration (Fig 6.C1) in terms of parameter *α*, again the higher parameter (*α* = 1.2) performed better for both a 40% coverage (5.77–6.53 steps) and an 80% coverage, with the difference increased even more (34.26–45.40 steps). Both parameters obtained significantly worse results than the processes without habituation, as can be clearly seen in Fig 6.C2. For a 40% network coverage, the duration increased for *α* = 1.2 by 74.85% and for *α* = 1.05 by 97.88%, whereas for an 80% coverage, the duration increased for these parameters by 262.12% and 379.92%, respectively.

Propagation probability (Fig 6.D1) was shown to be a much stronger factor influencing the duration of processes with habituation compared to without habituation. For an 80% coverage, processes without habituation took 69% longer with *PP* = 0.05 than with *PP* = 0.1 (11.91–7.01 steps). For the same coverage value, processes with habituation for a lower *PP* lasted 170% longer (58.14–21.52 steps). This is further evidence that, as time passes, the habituation process has an increasing effect on decreasing spreading efficiency, directly affecting the overall process’s duration.

The fewer nodes infected in a given step, the longer the entire process takes (Fig 6.D2). To achieve the assumed network contagion threshold, processes with habituation when *PP* = 0.05 take 388.16% longer than processes without the habituation effect; when *PP* = 0.1, the increase is 206.99%. A trend can be noted that a doubled *PP* causes a two-fold decrease; i.e., it is inversely proportional. Reaching a 40% network coverage where *PP* = 0.1 for processes with habituation takes only 37.32% longer than processes without habituation; when *PP* = 0.05, this percentage is 121.61%. Reaching a 40% network coverage where *PP* = 0.1 for processes with habituation takes only 37.32% longer than for processes without habituation; when *PP* = 0.05, this percentage is, again, 121.61% for processes with habituation.

The effect of the number of nodes that initiate the entire process is shown in Fig 6.E1. For coverage up to the 40% level, habituation-aware simulations with *SP* = 0.05 reach the assumed threshold faster than processes with a lower initial seed count, *SP* = 0.01. On the other hand, reaching the 80% threshold for the same processes takes longer with a higher *SP* = 0.05. This could imply that the initial high number of infected nodes results in a higher number of ineffective attacks, which, after the early rapid coverage of the network, results in a concomitant, faster decrease in the average responsiveness of the entire network, which slows down the process, making it more difficult for subsequent infections to occur at the same rate. Fig 6.E2 shows the increase in the duration of processes with the habituation effect relative to processes without it. Processes with the higher initial number of infected nodes lasted longer when they reached both a 40% and 80% network coverage. As in the previous graph, we can observe that the processes with habituation and a higher *SP* perform worse at the initial stage due to a decrease in responsiveness compared with processes without habituation and thus an *SP* value. Again, the decrease in average network responsiveness at earlier stages of the process slows down the spreading from the very beginning.

The effects of individual values of *τ* on the increase in the duration of the network coverage process are shown in Fig 6.F1. As assumed, increasing the value of *τ* promotes process acceleration. Processes with a *τ* value of 5 take about three times as long to reach the assumed thresholds than processes with a *τ* value of 25. Fig 6.F2 shows the increases in the duration of processes with habituation relative to processes without it. Again, it can be observed that the more rapidly the habituation process is, the more quickly the responsiveness of the network decreases, which is a simple factor that leads to fewer infected nodes in subsequent steps. This means more failed infection attempts, which in turn again causes the responsiveness to drop, and so on. For a *τ* value of 5 for a 40% network coverage, the process duration increases by 200%; for a threshold of 80%, the time increases by over 600%. As an extreme comparison, a *τ* of 25 reaches the 80% threshold with an increase of less than 200%.

## 6. Conclusions

Most earlier works related to repeated contacts within social networks and information diffusion processes have assumed an increased potential response with each repetition. From that perspective, each contact may increase a node’s interest in a discussed product or idea. As a result, the transmission rate increases.

From another perspective, each incoming message can be treated as a stimulus, and according to research related to habituation, the response to repeated stimuli decreases. However, a response also has the potential to recover if stimuli are not encountered over a certain timeframe. In this study, we evaluated the impact of habituation on spreading processes under an SI model with assumed repeated contact during the process.

Our experimental study revealed the existence of a relationship between process dynamics and selected habituation parameters. Many significant decreases in performance and the coverage of simulated processes were observed. We believe that failing to account for the habituation effect can result in significant performance degradations in real systems, despite the selection of appropriate seeds and individual process parameters to maximize impact. This maximization may turn out to be “lethal” for the intended effects of the campaign, as content overload will have the opposite effect.

One of the ways to avoid such situations may be the selection of an appropriate “dosage” of content so as not to lead to a decrease in responsiveness, which this study tries to prove by looking for causality in the habituation effect. The obtained results have several implications for practice and real campaigns. Spreading processes in social networks allows us to analyze the entire spectrum of events that affect the functioning of the world, including marketing, social and political campaigns, viral rumor marketing, and the entire flow of information, ending with the widely discussed current topic of the ongoing COVID-19 pandemic. The means of communication can generate different stimuli to change the user’s behavior or opinion. Maximizing contact or influence and seed choice were considered when analyzing such an impact. Although increasing the number of contacts may be effective for many scenarios, repeated unwanted messages, instead of the intended maximization, may have the opposite effect.

An interesting example may be the ongoing large-scale vaccination campaigns all around the world. After very rapid initial growth, vaccination rates quickly began to decrease, many countries have not yet reached their intended thresholds. At the same time, the number of people who were skeptical about vaccination increased. Information that annoys people the first time around, when repeated persistently, will increase the level of aversion in the recipient and increase the likelihood that they will discourage additional people in their environment. At the same time, a “latent” disinformation campaign is underway in a form similar to whisper marketing, in which information is infrequent but highly emotionally tinged.

Several directions of future work can be taken. First of all, the need for methods allowing us to decrease the negative impact of habituation of information diffusion performance is strongly suggested. Appropriate timing and campaign intensities can be adjusted using computational models. Another exciting topic is related to competing processes and the impact of habituation on their interactions.
