# Correction: Modeling the impact of the habituation effect on information spreading processes with repeated contacts under an SI model

**DOI:** 10.1371/journal.pone.0286690

**Published:** 2023-05-30

**Authors:** The PLOS ONE Staff

## Notice of republication

This article was republished on April 24, 2023, to correct an author’s name which was misspelled. The publisher apologizes for the errors. Please download this article again to view the correct version. The originally published, uncorrected article and the republished, corrected articles are provided here for reference.

## Supporting information

S1 File Originally published, uncorrected article. (PDF)

S2 File Republished, corrected article. (PDF)
